# Anal melanoma: a clinical challenge without therapeutic consensus

**DOI:** 10.1093/jscr/rjaf015

**Published:** 2025-01-22

**Authors:** Kevin J Fuentes-Calvo, Francisco E Alvarez-Bautista, Oscar Santes, Renata De Nora-Jiménez, Luis F Arias-Ruíz, Noel Salgado-Nesme

**Affiliations:** Department of General Surgery, Hospital Médica Sur, Puente de Piedra 150, 140550 Mexico City, Mexico; Department of General and Endoscopic Surgery, Hospital General Dr. Manuel Gea González, Calzada de Tlalpan 4800, 14080 Mexico City, Mexico; Department of Colorectal Surgery, Instituto Nacional de Ciencias Médicas y Nutrición Salvador Zubirán, Vasco de Quiroga 15, 14080 Mexico City, Mexico; Department of Internal Medicine, Hospital Médica Sur, Puente de Piedra 150, 140550 Mexico City, Mexico; Department of Anatomic Pathology, Fundación Clínica Médica Sur, Puente de Piedra 150, 140550 Mexico City, Mexico; Department of Colorectal Surgery, Instituto Nacional de Ciencias Médicas y Nutrición Salvador Zubirán, Vasco de Quiroga 15, 14080 Mexico City, Mexico; Universidad Nacional Autónoma de México, Avenida Universidad 3000, 04510 Mexico City, Mexico

**Keywords:** anal canal malignancy, anal cancer, anal melanoma, surgical treatment, local wide excision

## Abstract

Anal melanoma is a rare malignancy, accounting for 0.4% to 1.6% of all melanomas. Its atypical presentation, low incidence, and non-specific symptoms make it a challenging diagnosis, which can lead to delayed treatment with an unfavorable impact on clinical outcomes. Treatment should be multidisciplinary and may include surgical resection with adjuvant therapy, chemotherapy, and radiotherapy. We present the case of a male patient who presented to the emergency department due to foreign body sensation and transanal bleeding. The patient underwent an anal exploration under anesthesia, where a hyperpigmented canal-dependent tumor lesion with extension into the perianal skin was found. After a wide local excision, histopathological study confirmed the diagnosis of invasive nodular melanoma. The patient was discharged without complications for follow-up and management in the outpatient medical oncology clinic.

## Introduction

Anal melanoma, also known as anorectal melanoma, is a rare malignant neoplasm arising from the anorectal mucosa that accounts for ~0.4% to 1.6% of all malignant melanomas [1, 2]. The diagnosis of anal melanoma is often delayed, and the disease exhibits aggressive biological behavior, resulting in an unfavorable prognosis [3].

## Case report

A 83-year-old man with a history of systemic arterial hypertension presented to the emergency department with transanal bleeding, foreign body sensation in the anal region, and unintentional weight loss of 5 kg in 3 months. The physical examination revealed a hyperpigmented and prolapsed lesion within the anal canal, accompanied by residual bleeding and hyperpigmentation extending to the anterior quadrants of the perianal skin. Considering the macroscopic characteristics of the lesion, a multidisciplinary team, comprising a colorectal surgeon, oncologist, and radiologist, was assembled for comprehensive evaluation and management.

**Figure 1:**
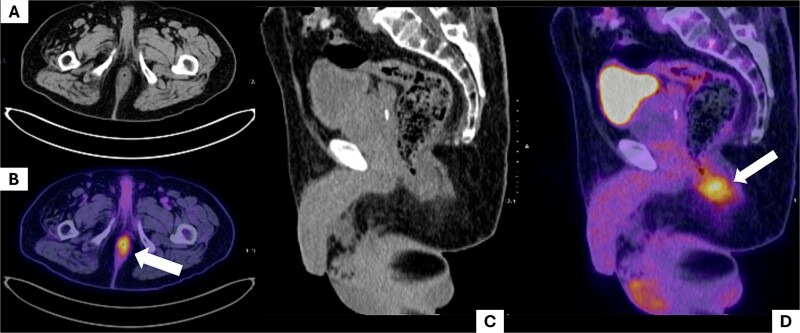
Positron emission tomography (PET-CT) scan. The images show the region corresponding to the anal canal. A and B show the affected anatomical region in axial slices. (A) Shows the anal canal with slight asymmetrical thickening predominantly on the left side, with no suspicious adenopathies; (B) shows uptake corresponding to the increase in local metabolism secondary to the tumor lesion (white arrow). C and D show sagittal slices of the pelvic region, where uptake is observed at the level of the anal canal (white arrow).

**Figure 2:**
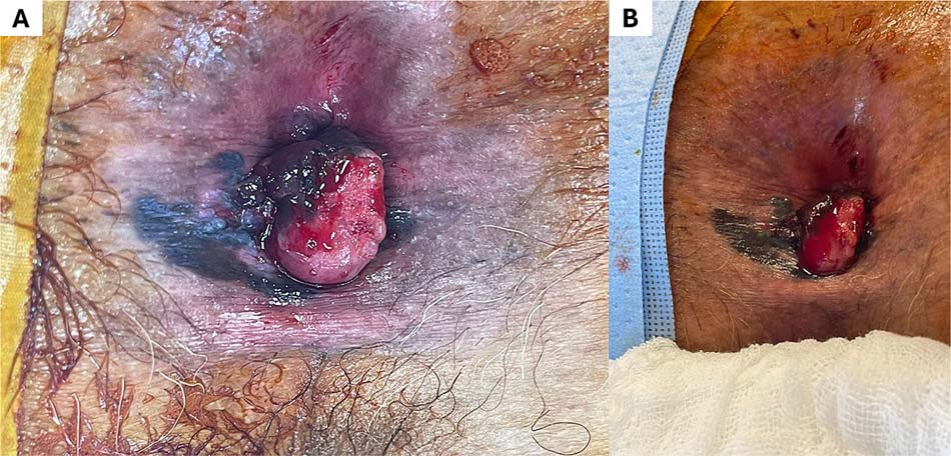
Lesion corresponding to anorectal melanoma. Observe in (A) and (B) the lesion with left lateral predominance, ulcerated surface, and irregular hyperpigmented borders.

**Figure 3:**
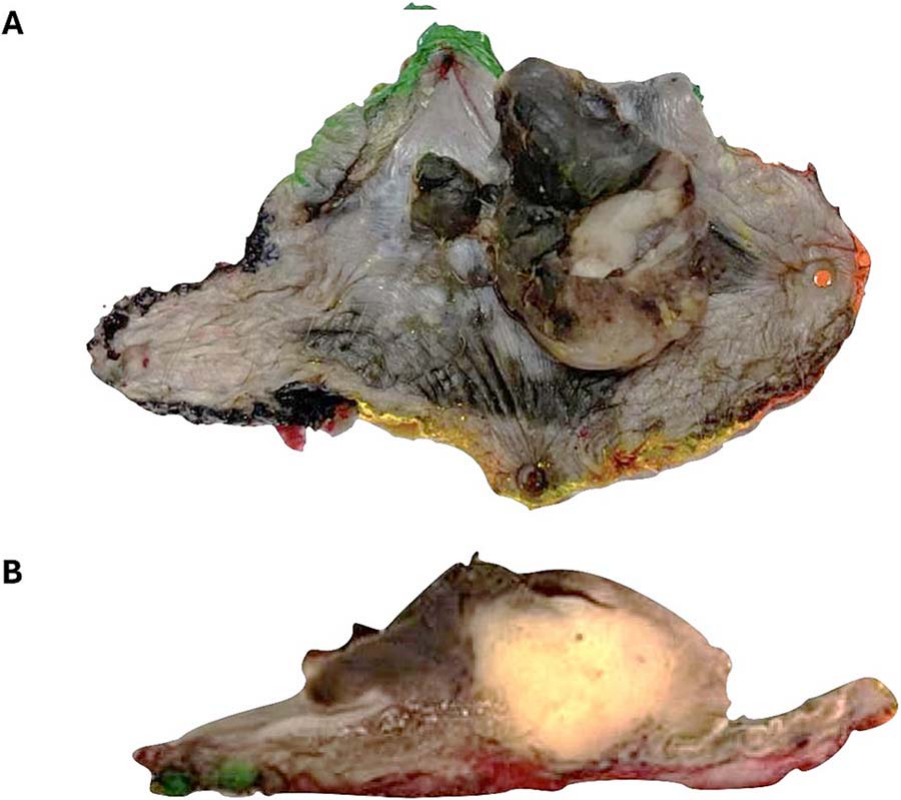
Wide local excision surgical specimen. (A) There is an exophytic tumor with irregular borders. (B) Nodular, submucosal, poorly demarcated tumor.

**Figure 4:**
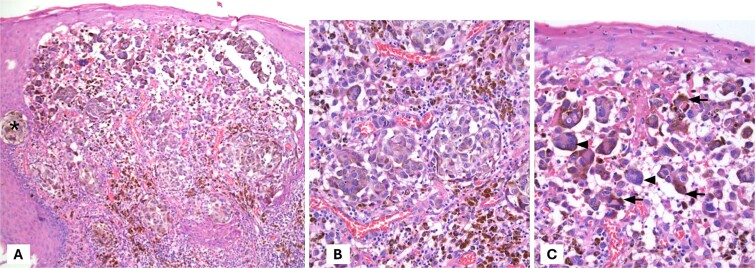
Hematoxylin and eosin staining. (A) Display of malignant neoplasm with solid growth pattern and forming nests (*). (B, C) At higher magnification, pleomorphic, polygonal cells with vesicular nuclei, prominent nucleolus, and abundant cytoplasm are observed. Note the pigment in the cytoplasm of the cells, which corresponds to melanin.

A positron emission tomography (PET-CT) scan demonstrated heightened metabolic activity at the level of the anal canal, without evidence of suspicious lymph nodes or distant metastases (Fig. 1). Surgical exploration of the anal canal revealed a hyperpigmented tumor-like lesion originating from the anal canal and involving the anterior quadrants of the perianal skin (Fig. 2). Subsequently, a wide local excision of the lesion was performed (Fig. 3*).* Histopathological examination confirmed the diagnosis of a poorly differentiated invasive nodular melanoma, measuring 3.3 × 2.8 cm, with a Clark level of IV and a Breslow depth of 6 mm (Fig. 4). The patient experienced an uneventful postoperative course and was discharged for follow-up and immunotherapy management in the outpatient medical oncology clinic.

## Discussion

Anorectal melanoma (AM) is a rare and aggressive mucosal melanocytic neoplasm that accounts for 1% of colorectal malignancies, < 0.5% of anal canal malignancies, and < 2% of melanomas. It represents the second most frequent subtype of mucosal melanoma after sinonasal melanoma [3, 4].

Anorectal melanoma predominantly affects individuals in the sixth to eighth decades of life, with a higher prevalence in women. The 5-year overall survival rates range from 14% to 30%, and the median overall survival is estimated to be between 9 and 19 months [5, 6]. Melanomas arise from melanocytes, which are found in the anal squamous zone and occasionally in the transition zone of the anorectal region. Malignant transformation of melanocytes in the anorectal mucosa is supposed to be associated with immunosuppression and oxidative stress [7].

Clinical presentation is often nonspecific, with symptoms such as transanal bleeding (84.9%), anal pain (68.7%), changes in bowel habits (28.5%), tenesmus (16.9%), and mucus secretion (6%) [3]. This nonspecific presentation can contribute to delayed diagnosis and increased potential for disease dissemination, accounting for the higher proportion of patients with regional or distant metastases at the time of diagnosis, compared to 11% observed in those with cutaneous melanomas [6]. Consequently, 7% to 20% of patients present with inguinal lymph node involvement at the time of anal melanoma diagnosis [4].

Surgery remains the cornerstone of treatment, with abdominoperineal resection (APR) and wide local excision (WLE) being the most commonly performed surgical interventions. In a recent meta-analysis of 303 patients, the 5-year overall survival rate was 23% for those undergoing APR and 32% for those undergoing WLE, with no statistically significant difference between the two groups. APR was associated with a local recurrence rate of 20.82%, while the recurrence rate after WLE was 47.04%. APR was associated with a significant reduction in local recurrence (OR 0.15, 95% CI 0.08–0.28, *P* < 0.00001) [8]. However, the patient’s general condition and disease stage should be considered when selecting the surgical approach, as APR carries a higher risk of morbidity. WLE may be considered for curative intent, as well as for palliation and improving the quality of life in cases where APR is not suitable [9, 10].

Currently, the role of adjuvant therapies, such as radiotherapy (including iliac and inguinal lymph node chains), chemotherapy, and immunotherapy, remains a subject of ongoing discussion. While these therapies may prolong disease-free survival, their impact on overall survival has not been definitively established [11, 12].

A multidisciplinary approach facilitates individualized treatment strategies based on the patient’s specific needs and clinical presentation. Further research is necessary to determine the true efficacy of emerging pharmacological therapies and their impact on survival, while carefully considering the potential for associated morbidity.

## Conclusion

Anal melanoma is a rare and aggressive malignancy characterized by delayed diagnosis due to nonspecific symptoms and late presentation, resulting in advanced-stage detection and a poor prognosis. Surgical intervention remains the mainstay of anal melanoma management, and may be complemented by adjuvant therapies. Further research is warranted to evaluate the efficacy and impact of these novel therapies on survival outcomes.

## References

[1] Chen H , Cai Y, Liu Y, et al. Incidence, surgical treatment, and prognosis of anorectal melanoma from 1973 to 2011: a population-based SEER analysis. Medicine 2016;95:e2770. 10.1097/MD.0000000000002770. 26886623 PMC4998623

[2] Mastoraki A , Schizas D, Ntella V, et al. Clinical evidence, diagnostic approach and challenging therapeutic modalities for malignant melanoma of the anorectum. ANZ J Surg 2021;91:276–81. 10.1111/ANS.16497. 33369807

[3] Paolino G , Podo Brunetti A, De Rosa C, et al. Anorectal melanoma: systematic review of the current literature of an aggressive type of melanoma. Melanoma Res 2024;34:487–96. 10.1097/CMR.0000000000001003. 39361336 PMC11524631

[4] Meguerditchian AN , Meterissian SH, Dunn KB. Anorectal melanoma: diagnosis and treatment. Dis Colon Rectum 2011;54:638–44. 10.1007/DCR.0B013E31820C9B1B. 21471767

[5] Bullard KM , Tuttle TM, Rothenberger DA, et al. Surgical therapy for anorectal melanoma. J Am Coll Surg 2003;196:206–11. 10.1016/S1072-7515(02)01538-7. 12595048

[6] Nagarajan P , Piao J, Ning J, et al. Prognostic model for patient survival in primary anorectal mucosal melanoma: stage at presentation determines relevance of histopathologic features. Mod Pathol 2020;33:496–513. 10.1038/S41379-019-0340-7. 31383963

[7] Saadaat R , Saifullah AMA, Rasool EE, et al. Primary malignant melanoma of rectum: a rare case report. Int J Surg Case Rep 2023;104:107942. 10.1016/J.IJSCR.2023.107942. 36801769 PMC9957745

[8] Temperley HC , O’Sullivan NJ, Keyes A, et al. Optimal surgical management strategy for treatment of primary anorectal malignant melanoma-a systematic review and meta-analysis. Langenbecks Arch Surg 2022;407:3193–200. 10.1007/S00423-022-02715-1. 36331615

[9] Bello DM , Smyth E, Perez D, et al. Anal versus rectal melanoma: does site of origin predict outcome? Dis Colon Rectum 2013;56:150–7. 10.1097/DCR.0B013E31827901DD. 23303142 PMC3543771

[10] Falch C , Mueller S, Kirschniak A, et al. Anorectal malignant melanoma: curative abdominoperineal resection: patient selection with 18F-FDG-PET/CT. World J Surg Oncol 2016;14:185. 10.1186/S12957-016-0938-X. 27422527 PMC4947294

[11] Terada R , Ito S, Kobayashi M, et al. Anorectal melanoma: successful treatment by surgical excision and combination chemoimmunotherapy. Hepatogastroenterology 2002;49:1545–8. 12397731

[12] Nam S , Kim CW, Baek SJ, et al. The clinical features and optimal treatment of anorectal malignant melanoma. Ann Surg Treat Res 2014;87:113–7. 10.4174/ASTR.2014.87.3.113. 25247163 PMC4170582

